# Pelvic ring injury in the elderly: Fragile patients with substantial mortality rates and long-term physical impairment

**DOI:** 10.1371/journal.pone.0216809

**Published:** 2019-05-28

**Authors:** Hester Banierink, Kaj ten Duis, Rob de Vries, Klaus Wendt, Erik Heineman, Inge Reininga, Frank IJpma

**Affiliations:** 1 University of Groningen, University Medical Center Groningen, Department of Trauma Surgery, Groningen, The Netherlands; 2 Emergency Care Network Northern Netherlands (AZNN), Northern Netherlands Trauma Registry, Groningen, The Netherlands; 3 University of Groningen, University Medical Center Groningen, Department of Surgery, Groningen, The Netherlands; Medical College of Wisconsin, UNITED STATES

## Abstract

**Background:**

Pelvic ring injuries in the elderly often occur after low-energy accidents. They may result in prolonged immobilization, complications and an intense rehabilitation process. The aim of this study was to assess mortality, physical functioning and quality of life (QoL) in elderly patients with pelvic ring injuries.

**Methods:**

A cross-sectional study was performed including all elderly patients (≥ 65 years) admitted for a pelvic ring injury between 2007-2016. Mortality and survival were evaluated and patient reported outcome measures (PROMs) were used to assess physical functioning (SMFA) and QoL (EQ-5D). These were compared to age-matched normative data from the general Dutch population.

**Results:**

A total of 153 patients, with a mean age of 79 years (SD 8) at the time of injury, were included in this study. The mortality rate was 20% at 30 days, 27% at 1 year and 41% at 3 years of follow-up. All six patients with a type C fracture died within 30 days. Analyses of the 153 patients showed that increasing age, fracture type C and Injury Severity Score (ISS) were all independent risk factors for mortality. Eventually, after excluding patients that died (N = 78) or were unable to contact (N = 2), 73 patients were eligible for follow-up, of which 53 patients (73%) responded. Mean Short Musculoskeletal Function Assessment (SMFA) scores were respectively 67.4 (function index), 65.2 (bother index), 66.5 (lower extremity), 60.4 (activities of daily living) and 68.2 (emotion). Mean EuroQuol-5D (EQ-5D) score was 0.72. Overall, physical functioning and quality of life were significantly decreased in comparison with normative data from the general population.

**Conclusion:**

Elderly people who sustain a pelvic ring injury should be considered as a fragile population with substantial mortality rates. The patients who survived demonstrated a substantially lower level of physical functioning and quality of life in comparison with their age-matched peers from the general population.

**Level of evidence:**

IV, therapeutic study.

## Introduction

The elderly population (≥ 65 years of age) has rapidly increased over the last few decades and it is predicted that this growth will continue in the future. In the Netherlands, the elderly population will grow from 2.7 million in 2012 to 4.7 million in 2041 [1]. One-third of all fractures and 73% of all pelvic injuries occur in the elderly [2]. Although the overall incidence of a pelvic ring injury is estimated at 20-37/100,000 per year [3], the incidence rises to 92/100,000 per year for the population aged over 65 years [4].

The elderly population is vulnerable as a result of age-related reduced physical condition, pre-existing comorbidities, limited rehabilitation capacity and decreased coping mechanisms. Although most fractures are isolated and stable, the ability of the elderly to mount a physiologic response is limited and hence high morbidity and mortality rates are reported [5]. The majority of pelvic ring injuries in this population is caused by low-energy mechanisms like a fall from standing position, often resulting in AO type A fractures [6–8], that are considered stable fractures with an intact posterior arch involving innominate bone avulsion, iliac wing, pubic rami, transverse sacral or coccyx fractures [9].

The rehabilitation to independent mobilization for this group is of utmost importance. This determines whether someone could regain its autonomy and will be able to participate in social activities. Yet, it frequently occurs that elderly patients with a pelvic ring injury end up in nursing homes and are not able to return to their own household [10]. They are prone to complications like decubitus, pneumonia and urinary tract infections [11]. Moreover, long-term permanent disabilities can affect their daily physical functioning and quality of life [12]. Hence, optimal treatment of pelvic ring injuries remains challenging, requiring a timely multidisciplinary approach.

In the elderly patients with pelvic ring injuries, mortality has often been studied intensively, while physical functioning and quality of life have hardly been assessed by means of patient reported outcome measurements (PROMs). We hypothesized that factors like comorbidity, fracture type, injury severity and age might influence mortality following pelvic ring injuries in the elderly. Moreover, physical functioning of these patients may be decreased compared to that of the general population. Hence, the aim of this study was to assess risk factors for mortality, as well as to provide an overview of physical functioning and quality of life of elderly patients after pelvic ring injuries.

## Patients and methods

### Patients

A cross-sectional study was performed. Elderly patients (≥ 65 years of age) who were treated for a pelvic ring injury at the Department of Trauma Surgery of the University Medical Center Groningen (UMCG) between January 2007 and January 2016 were included. For all patients, the life status (alive or date of death) and the current contact details were verified in the Dutch population registry. All patients alive at the time of the study were contacted and asked to complete questionnaires in order to assess long-term physical functioning and quality of life. Patients with cognitive disorders were excluded from follow-up with the questionnaires. The local Medical Ethical Review Board reviewed the methods employed and waived further need for approval (METc 2016.385).

### Methods

**Fig 1:**
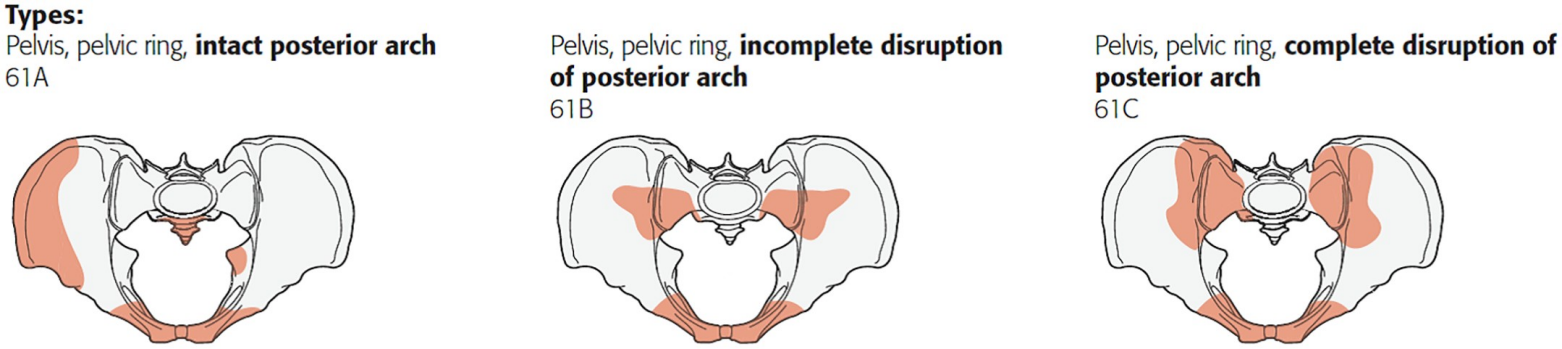
Types of pelvic ring injuries [9].

The patients’ demographics and clinical characteristics concerning injury mechanism and fracture type were collected by reviewing their medical and operation records. Injury mechanisms were divided into low- or high-energy trauma. Low-energy trauma mainly consists of a low-energy fall, which is defined by the Dutch Trauma Registry (DTR) [13] as a fall below two-to-three times the body length. Injury characteristics in terms of the Abbreviated Injury Scale (AIS) and Injury Severity Score (ISS) [14,15] were retrieved from the DTR. The AIS is an anatomically based global injury severity scoring system that helps to classify the injury on the level of severity, based upon different body regions. The scores vary from 1 (minor) to 6 (currently not treatable). The AIS can be used to calculate the ISS, which is sum of squares of the three highest AIS scores of three different body regions and can range from 1 to 75, in which 75 means that the chance of survival is extremely low. The Charlson comorbidity index score (CCI) [16] was calculated to evaluate the pre-injury condition. The CCI provides a simple and valid method of estimating risk of death from comorbid disease by scoring the severity of the comorbid conditions and adding up the scores on a scale from 1–6, with 1 extra point for each decade above 40 years of age. Two senior trauma surgeons with ample experience in pelvic fracture surgery assessed the radiographic images (plain anteroposterior, inlet and outlet radiographs and computerized tomography scans) of all the patients and classified the pelvic ring injuries into type A, B and C injuries, according to the Tile/AO classification (Fig 1) [9,17].

### Complications, mortality and survival

Demographics and injury characteristics of patients still alive at follow-up were compared with those of patients that had died. It was evaluated whether age, sex, injury mechanism (low- vs. high-energy trauma), fracture type, complications, CCI and ISS were independent mortality risk factors and whether effect modification existed. Moreover, survival was analysed in three age groups (age 65–75, 76–85, and >85). Mortality rates were compared to those of the general Dutch population, based on the numbers provided by the national Central Agency for Statistics [18].

### Functional outcome instruments

Physical functioning was measured with the Dutch version of the Short Musculoskeletal Function Assessment (SMFA-NL), consisting of the two original indices (function and bother) [19] and four additional subscales (lower extremities, upper extremities, daily activities and emotion) [20]. The 46 items are scored on a 5-item Likert scale, ranging from 1 (poor function) to 5 (good function). Scores are calculated by summing up the individual items and transforming scores on a range from zero to 100, with higher scores indicating better function. Missing items in the SMFA were handled according to the instruction manual of this questionnaire. In case less than 50% of the answers were missing in any category of the function index, the mean value of that category was substituted for the missing items. If answers were missing in the bothersome index, patients were omitted from the analysis of this index. Quality of life was assessed with the EuroQol 5D (EQ-5D) [21], which screens five health levels (mobility, self-care, daily activities, pain/inconvenience and fear/depression) and is expressed as a score from -0.329 (worst condition) to 1 (best QoL). Both the SMFA and EQ-5D scores of the patients in this study were compared to the normative data of the age-matched general Dutch population [22,23]. The EQ-5D instruction manual does not provide information on how to handle missing items. Therefore, in case one or more items were missing, data of these patients were omitted from further analysis.

### Statistical analysis

Demographic and clinical data are presented as means and standard deviations (SD) for the continuous variables and as percentages for categorical variables. Median and interquartile range (IQR) are presented for non-Gaussian distributions. Either independent samples t-test or Mann-Whitney U Test were performed accordingly to detect mean differences between the groups that had deceased or not. Categorical variables were evaluated using the chi-squared test. Survival was analysed using a Kaplan-Meier curve. Additionally, independent predictors for mortality were analysed by using a multivariate backward cox regression analysis with the removal p-value set at 0.157. The variables age at time of injury, low- vs. high-energy trauma and ISS were checked for possible effect modification. A non-response analysis was performed to evaluate differences between responders and non-responders. Difference in functional outcome and QoL (SMFA-NL and EQ-5D) between the study population and the age-matched general Dutch population was assessed by using the independent samples T-test. The level of significance was defined at p < 0.05. The data were analysed using the IBM SPSS software, version 23.0 for Windows (IBM Corporation, Armonk, NY).

## Results

### Patient and injury characteristics

The data concerning patient and injury characteristics are presented in Table 1. A total of 153 elderly patients with pelvic ring injuries were identified over a study period of 9 years (January 2007 until January 2016). Age ranged from 65 to 100 years at the time of injury (mean (SD) 79 (8)) and mean follow-up was five years after injury. Forty-five patients were men (29%). The majority of the pelvic ring injuries were classified as AO type A (66%) injuries. Most patients (63%) sustained low-energy traumas and median ISS was 9 (range 4–59). Four patients needed a trauma laparotomy and five patients underwent angio-embolization. In the whole study cohort, 35 complications occurred within 30 days in 25 patients (16%), the majority being delirium (N = 12) and pneumonia (N = 8).

**Table 1:** Baseline characteristics of patients alive and deceased 30 days after injury.

	All patients (N = 153)	Patients deceased within 30 days after injury (N = 31)	Patients alive after 30 days (N = 122)	P-value*
Age at time of injury median (IQR)	79 (71–84)	79 (79–84)	80 (71–84)	0.57
Male	45 (29)	13 (42)	32 (26)	0.12
**Low-energy trauma**	97(63)	4 (13)	93 (76)	**<0.001**
**High-energy trauma**	56 (37)	27 (87)	29 (24)	**<0.001**
Fall from height	7 (13)	1 (3)	6 (5)	-
Crush injury	1 (2)	-	1 (1)	-
One-sided motor vehicle/ motorcycle injury	4 (7)	2 (7)	2 (1)	-
Pedestrian/cyclist vs. motor vehicle/motorcycle	27 (48)	15 (48)	12 (10)	-
Motor vehicle/motorcycle vs. motor vehicle/motorcycle	16 (29)	8 (26)	8 (7)	-
Shot injuries	1 (2)	1 (3)	-	-
**Fracture classification**				**0.001**
Type A	101 (66)	12 (39)	89 (73)	-
Type B	42 (28)	11 (36)	31 (25)	-
Type C	6 (4)	6 (19)	-	-
No Classification**	4 (2)	2 (6)	2 (2)	-
**Complications <30 days**	35 (23)	2 (7)	23 (19)	**0.02**
Delirium	12 (34)	1 (3)	11 (9)	-
Pneumonia	8 (24)	1 (3)	7 (6)	-
Urinary infection	6 (17)	-	6 (5)	-
Urinary system	3 (9)	-	3 (2)	-
Wound infection	1 (3)	-	1 (1)	-
Infection (other)	2 (5)	1 (3)	1 (1)	-
Lung embolism/DVT	2 (5)	-	2 (1)	-
Bleeding	1 (3)	-	1 (1)	-
Nerve injury	-	-	-	-
Unknown (e.g. patient was transferred to another hospital/institution)	6 (4)	-	6 (5)	-
**Highest AIS pelvis** median (IQR)	2 (2–3)	3 (3–3)	2 (2–2)	**<0.001**
**ISS** median (IQR)	9 (4–25)	34 (34–45)	5 (5–13)	**<0.001**
**ISS >15**	55 (36)	29 (93)	26 (21)	**<0.001**
CCI median (IQR)	5 (4–6)	5 (5–6)	5 (5–7)	0.41

Numbers are expressed in N with the percentage in parentheses unless otherwise specified

* Statistically significant results are in bold

** Classification could not be performed due to lack of imaging

The majority of the study population was treated conservatively (N = 141, 92%), whereas only 12 patients (8%) were treated operatively with respectively plate fixation (N = 6), an external fixator (N = 2), SI screws (N = 1), a combination with plate fixation and SI screws (N = 2), or a combination with plate fixation and an external fixator (N = 1). Conservative treatment of pelvic ring injuries consisted of early mobilization with weight bearing as tolerated in combination with appropriate pain medication. Eventually, 31 patients were discharged to a nursing home.

Fifteen patients (10%) died at the day of the injury and a total of thirty-one patients (20%) died within the first 30 days after the injury. All six patients with type C injuries had died within 30 days after the injury. Comparison of the group that had died within the first 30 days after the injury to the group that survived this critical period revealed significant differences in injury mechanism (low- or high-energy trauma), fracture type (A, B or C), complications, AIS and ISS (Table 1).

### Survival analysis

**Fig 2:**
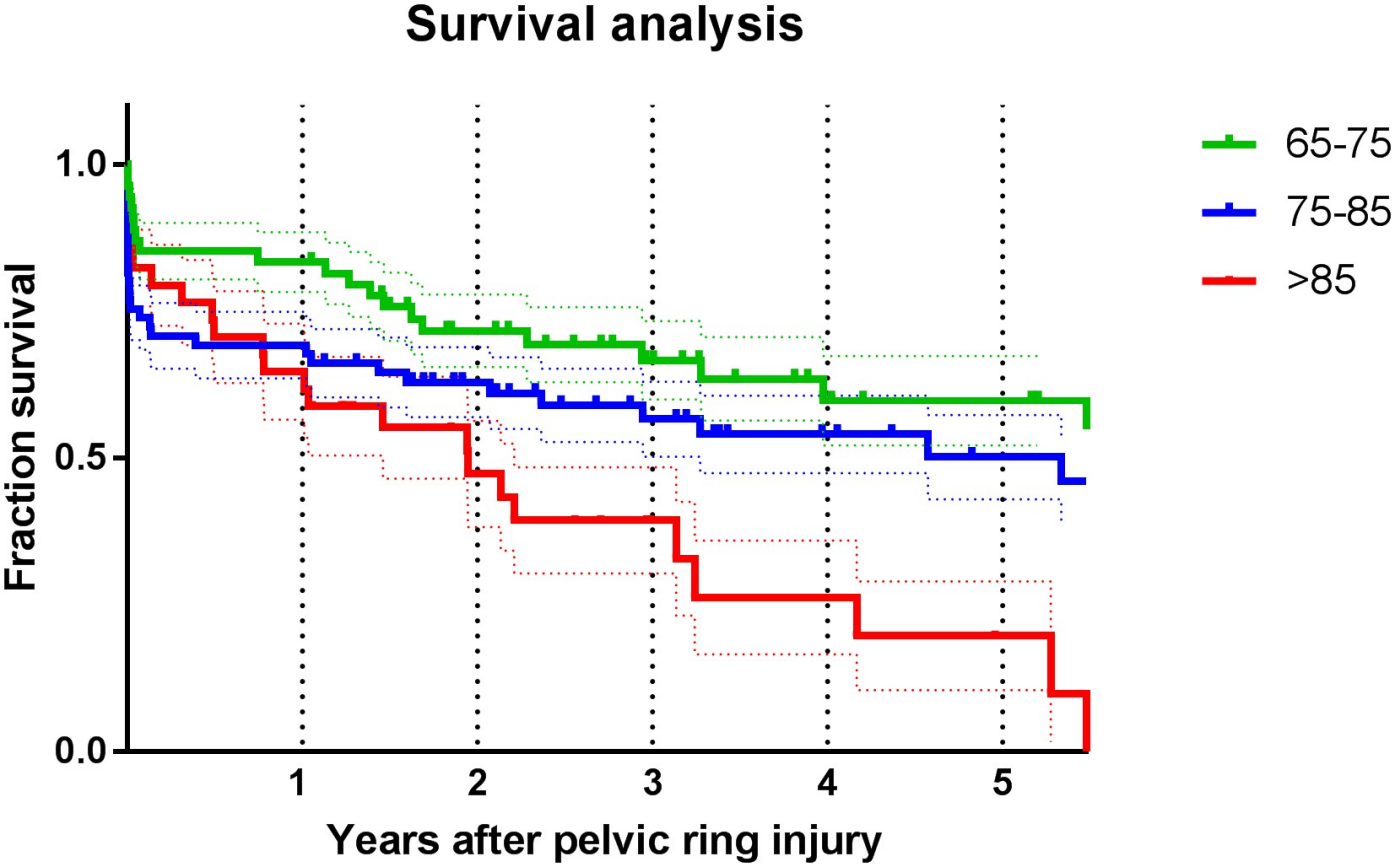
Kaplan-Meier survival curve indicating survival in years according to age at the time of injury.

A total of 41 patients (27%) died within a year and 63 patients (41%) within 3 years after the injury. Fig 2 demonstrates the survival of the elderly patients divided into three age-groups with survival rates decreasing for patients aged 75–85 years, and even more for those aged >85 years, compared to patients aged 65–75 years at the time of injury. There was a significant difference in one-year mortality (P = 0.007) between the three age groups. Table 2 demonstrates the mortality rates of the three age groups from year one up to year five after the injury (rows 2, 4 and 6) and the mortality rates from the general Dutch population (rows 3, 5 and 7). This table demonstrates excessive differences in mortality after sustaining a pelvic ring injury compared to the general population.

**Table 2:** Cumulative percentages of deceased patients from the study population and the Dutch population according to age at time of injury.

	N	Year 1	Year 2	Year 3	Year 4	Year 5
**65–75**	54	17%	39%	56%	70%	74%
65–75 (NL)*		2%	3%	5%	7%	8%
**76–85**	65	31%	49%	63%	75%	82%
76–85 (NL)*		5%	11%	16%	21%	27%
**>85**	34	35%	65%	82%	88%	94%
>85 (NL)*		15%	29%	42%	53%	62%

* Mortality rates of the general Dutch population (Central Agency for Statistics) [18].

### Mortality risk factors

Cox regression analysis was performed to assess independent risk factors for mortality. No effect modification existed. The analysis showed that higher age at time of injury, pelvic fracture type C and higher ISS were shown to be independent risk factors for mortality (Table 3). There was a 7% increase in the odds of dying with every year of increasing age. Moreover, patients with type C fractures were almost five times more likely to die than patients with type A fractures. Finally, the odds of the patients dying increased by 6% with every point increase in ISS. Higher Charlson Comorbidity Index tended to have an effect on mortality as well, although not statistically significant (P = 0.07).

**Table 3:** Multivariate cox regression analysis on mortality.

	N	HR	95% CI		p-value*
Final multivariate model					
**Age at time of injury (years)**	153	1.07	1.03	1.10	**<0.001**
Fracture type**	153				
Type B		0.75	0.39	1.45	0.39
Type C		4.70	1.54	14.40	**0.007**
**ISS**	153	1.06	1.04	1.09	**<0.001**
**CCI**	153	1.13	0.99	1.28	0.07

* Statistically significant results are in bold.; HR: hazard ratio; ISS: Injury Severity Score; CCI: Charlson comorbidity index.

** Reference category: fracture type A.

### Follow-up by means of PROMs

Of the 153 patients, 51% (N = 78) of the patients had died at long-term follow-up and two patients were living abroad and could therefore not be contacted, leaving 73 patients eligible for follow-up by means of patient-reported outcome measures. A total of 53 patients (73%) responded at a mean follow-up of 3.4 ± 2.7 years after the pelvic ring injury. The other 20 patients (27%) declined to participate or did not respond. A non-response analysis showed differences in the proportion of pelvic fracture types; a higher proportion of patients with a type B injury responded (P = 0.01). Moreover, patients with higher ISS were more likely to respond (P = 0.002). No other differences were found between the responders and non-responders.

### Physical functioning and quality of life

Overall, patients with pelvic injuries reported moderate limitations with respectively a mean of 67.4 on the function index, 65.2 on the bother index, 66.5 on the lower extremity, 60.4 on the ADL (activities of daily living) and 68.2 on the emotion subscale of the SMFA (Table 4). Concerning the lower extremity subscale of the SMFA, patients indicated having the most problems with climbing stairs and with bending and kneeling down. Patients who had sustained any type of pelvic ring injury reported a reasonable QoL (Table 4) with a mean EQ-5D score of 0.72. The comparison of SMFA and EQ-5D scores with the age-matched normative data from the general Dutch population revealed significant differences regarding all parts of the SMFA as well as the EQ-5D, meaning that physical functioning and quality of life in an elderly patient with a pelvic ring injury was significantly decreased (Table 4).

**Table 4:** Outcomes on the SMFA-NL and EQ-5D.

	Study population	General Dutch population	P-value*
**SMFA**			
**Function Index**			
Mean ± Std.	67.4 ± 29.4	87.1 **±** 13.5	**0.001**
**Bother Index**			
Mean ± Std.	65.2 ± 26.7	84.7 ± 18.7	**<0.001**
**Lower extremity**			
Mean ± Std.	66.5 ± 31.2	86.4 ± 14.8	**0.001**
**ADL**			
Mean ± Std.	60.4 ± 32.0	86.0 ± 17.3	**<0.001**
**Emotion**			
Mean ± Std.	68.2 ± 20.1	80.2 ± 17.1	**<0.001**
**EQ5D**			
Mean ± Std.	0.72 **±** 0.277	0.87 ± 0.170	**<0.001**

* Statistically significant results are in bold.;

ADL: activities of daily living

## Discussion

Elderly patients who sustain a pelvic ring injury are fragile and prone to complications, high rates of mortality as well as physical impairment and decreased quality of life (QoL). This study revealed high mortality rates (up to 41% after 3 years) among the elderly patient with a pelvic ring injury and demonstrated that survival rate decreased as the patient’s age and ISS increased and when pelvic fracture type is more severe. Moreover, elderly patients demonstrated a substantially lower level of physical functioning and quality of life 3 years after pelvic ring injury, in comparison to their peers from the general Dutch population.

The mortality rates of elderly people who had sustained a pelvic ring injury were high, namely 10% at the day of the injury, 20% within 30 days, 27% within a year and 41% at 3 years of follow up. Morris et al. reported a comparable one-year mortality rate [8]. Although Balogh et al. found a slightly lower one-year mortality rate, it was comparable at 23% [24]. With 12.9%, Bible et al. [25] found a lower one-year mortality rate. However, they only included isolated pelvic fractures with posterior ring involvement, whereas our study included all types of pelvic ring injuries. Moreover, the 1-year mortality rates in elderly who sustained a pelvic ring injury (27% in this study) seem comparable with elderly with intertrochanteric or femoral neck fractures (21–23% according to a review of RCTs by Mundi et al.) [26].

The present study demonstrated a significant difference in one-year mortality between the different age groups (65–75, 76–85 and >85 years of age), showing that the patients aged >85 had an increased risk of dying. Moreover, this study showed that the mortality rates of patients with pelvic ring injuries is substantially higher compared to the mortality rates of their age-matched peers from the general Dutch population. This emphasises the fragility of this patient population, although it is interesting to speculate on whether the increased mortality is because of the injury or whether the injury itself is a sign of physical and general systems decline. De Vries et al. showed that elderly patients sustaining a polytrauma have an increased risk of dying compared to younger patients, even though the severity of the injury is comparable [27]. Given these numbers, the high impact of a pelvic ring injury in the elderly, with often pre-existing comorbidity, limited rehabilitation capacity and coping mechanisms, should not be underestimated.

In this study, high age at time of injury, type C fractures and ISS were shown to be independent mortality risk factors. A recent study by Verbeek et al. revealed age as the most important independent predictor for in-hospital mortality after any type of pelvic injury [28]. Not surprisingly, Forni et al., who evaluated predictive factors for 30-day mortality in geriatric patients with hip fractures, corroborated that advancing age is an independent risk factor for mortality [29]. In addition, several studies found older age, increased comorbidity, lower pre-fracture function, and cognitive impairment to be associated with higher three to six month mortality following surgically treated hip fractures as described in an extensive systematic review of the literature over the past decades [30].

Most studies of pelvic ring injuries in the elderly focused on mortality rates, but data about (the recovery of) physical functioning and quality of life of the survivors is hardly available. Schmitz et al. evaluated quality of life in patients aged 60 years and older after pelvic ring injuries and found a significant decrease compared to a reference population [12]. However, no data on physical functioning was published. In studies that focused on geriatric hip fractures, outcomes in terms of quality of life and physical functioning were sparsely assessed and consequently no real conclusions could be drawn [30]. Our study showed that both the long-term physical functioning as well as quality of life at a mean follow-up of 3.4 years after pelvic ring injury were significantly decreased when compared to the age-matched normative data from the general Dutch population. This indicates that not only the elderly show signs of fragility in terms of high mortality rates shortly after the injury, long-term effects of the injury may also reduce the patients physical functioning and quality of life. In order to improve the latter, physicians could for instance focus on a multidisciplinary approach, consulting a geriatrician, keeping a close eye on nutritional status and encourage early mobilisation under the direct control of a physiotherapist.

Thirty-one percent of the patients in this study was discharged to a nursing home. This is in concordance with previous research evaluating patients sustaining a pubic rami fracture [7], who were less likely to return to their original place of domicile. Another study by Studer et al. found that 43.4% of the elderly patients with a pubic rami fracture were institutionalized after one year [31]. Van Dijk et al. evaluated 99 patients with pelvic ring injuries and concluded that 33% of the patients needed temporary or permanent admission to a nursing home [32]. This underlines that decreased physical functioning as a result of the pelvic ring injury has a significant personal as well as societal impact.

This study has a retrospective character and is therefore susceptible to the inherent limitations such as the absence of baseline PROMs concerning the patients’ physical health prior to the injury. Moreover, although 73% of the patients in follow-up with questionnaires responded, this is only 35% of the total elderly population in our study due to high mortality rates. Another subject of discussion could be the generalization of the results because of the single centre study design. Yet, to the best of our knowledge, this is one of the few studies that evaluated both mortality and long-term functional outcome of elderly patients with a pelvic ring injury. Most studies on pelvic ring injuries in the elderly focused solely on complication and mortality rates. However, in our study, the patients’ own perception with regard to physical functioning and quality of life had a central role. The used patient-reported outcomes measures EQ-5D and SMFA-NL complement each other and are both valid and reliable questionnaires that provide a generalized physical functioning and quality of life outcome score. Moreover, using these PROMs enabled us to compare the results with age-matched normative data from the general Dutch population. Other strengths of this study are the long follow-up period and the comparison of mortality data to that of the general Dutch population.

In conclusion, elderly patients with pelvic ring injuries are fragile patients with high risks of mortality and decreased functional outcome compared to their peers from the general population. High age at the time of an accident, severity of the pelvic ring injury (type C) and ISS are all independent mortality risk factors. By highlighting the absolute numbers regarding mortality, physical functioning and quality of life among a large cohort of elderly who sustained a pelvic ring injury, we hope that physicians will be aware of the vulnerability of these patients and pay attention to interventions, like a multidisciplinary approach, optimal nutrition and early mobilization, which may benefit the injured elderly person.

## Supporting information

S1 File Short Musculoskeletal Function Assessment. (PDF)

S2 File Short Musculoskeletal Function Assessment—Dutch version. (PDF)

S3 File EQ-5D-5L. (PDF)

S4 File EQ-5D-5L—Dutch version. (PDF)

S5 File Anonymous dataset. (SAV)
