# Supplementary material for: Pelvic ring injury in the elderly: Fragile patients with substantial mortality rates and long-term physical impairment
Source: PLoS One. 2019 May 28;14(5):e0216809. doi: 10.1371/journal.pone.0216809 (PMC6538139; doi:10.1371/journal.pone.0216809)
Supplement: S1 File — (PDF) [file pone.0216809.s001.pdf]

Optimal Performance Physical Therapy

Short Musculoskeletal Function Assessment

Name: ____________________________ Signature: _________________ Date: ____________

Instructions: We are interested in finding out how you are managing with your injury or arthritis this week. We would like to know about any problems you may be having with your daily activities because of your injury or arthritis. Please answer each question by putting a check in the box corresponding to the choice that best describes you.

These questions are about how much difficulty you may be having this week with your daily activities because of your injury o arthritis.

		Not at all Difficult A	A Little Difficult B	Moderately Difficult C	Very Difficult D	Unable to do E
**1.**	How difficult is it for you to get in or out of a low chair?	☐	☐	☐	☐	☐
**2.**	How difficult is it for you to open medicine bottles or jars?	☐	☐	☐	☐	☐
**3.**	How difficult is it for you to shop for groceries or other things?	☐	☐	☐	☐	☐
**4.**	How difficult is it for you to climb stairs?	☐	☐	☐	☐	☐
**5.**	How difficult is it for you to make a tight fist?	☐	☐	☐	☐	☐
**6.**	How difficult is it for you to get in or out of the bathtub or shower?	☐	☐	☐	☐	☐
**7.**	How difficult is it for you to get comfortable to sleep?	☐	☐	☐	☐	☐
**8.**	How difficult is it for you to bend or kneel down?	☐	☐	☐	☐	☐
**9.**	How difficult is it for you to use buttons, snaps, hooks, or zippers?	☐	☐	☐	☐	☐
**10.**	How difficult is it for you to cut your own fingernails?	☐	☐	☐	☐	☐
**11.**	How difficult is it for you to dress yourself?	☐	☐	☐	☐	☐
**12.**	How difficult is it for you to walk?	☐	☐	☐	☐	☐
**13.**	How difficult is it for you to get moving after you have been sitting or lying down?	☐	☐	☐	☐	☐
**14.**	How difficult is it for you to go out by yourself?	☐	☐	☐	☐	☐
**15.**	How difficult is it for you to drive?	☐	☐	☐	☐	☐
**16.**	How difficult is it for you to clean yourself after going to the bathroom?	☐	☐	☐	☐	☐
**17.**	How difficult is it for you to turn knobs or levers (for example, to open doors or to roll down car windows)?	☐	☐	☐	☐	☐
**18.**	How difficult is it for to write or type?	☐	☐	☐	☐	☐
**19.**	How difficult is it for you to pivot?	☐	☐	☐	☐	☐
**20.**	How difficult is it for you to do your usual physical recreational activities, such as bicycling, jogging, or walking?	☐	☐	☐	☐	☐
**21.**	How difficult is it for you to do your usual leisure activities, such as hobbies, crafts, gardening, card-playing, or going out with friends?	☐	☐	☐	☐	☐
**22.**	How much difficulty are you having with sexual activity?	☐	☐	☐	☐	☐
**23.**	How difficult is it for you to do light housework or yard work, such as dusting, washing dishes, or watering plants?	☐	☐	☐	☐	☐
**24.**	How difficult is it for you to do heavy housework or yard work, such as washing floors, vacuuming, or mowing lawns?	☐	☐	☐	☐	☐
**25.**	How difficult is it for you to do your usual work, such as a paid job, housework, or volunteer activities?	☐	☐	☐	☐	☐

These next questions ask how often you are experiencing problems this week because of your injury or arthritis.

	None of the Time A	A Little of the Time B	Some of the Time C	Most of the Time D	All of the Time E
**26.** How often do you walk with a limp?	☐	☐	☐	☐	☐
**27.** How often do you avoid using your painful limb(s) or back?	☐	☐	☐	☐	☐
**28.** How often does your leg lock or give way?	☐	☐	☐	☐	☐
**29.** How often do you have problems with concentrating?	☐	☐	☐	☐	☐
**30.** How often does doing too much in one way affect what you do the next day?	☐	☐	☐	☐	☐
**31.** How often do you act irritable toward those around you(for example, snap at people, give sharp answers, or criticize easily)?	☐	☐	☐	☐	☐
**32.** How often are you tired?	☐	☐	☐	☐	☐
**33.** How often do you feel disabled?	☐	☐	☐	☐	☐
**34.** How often do you feel angry or frustrated that you have this injury or arthritis?	☐	☐	☐	☐	☐

These questions are about how much you are bothered by problems you are having this week because of your injury or arthrit

	Not at All Bothered A	A Little Bothered B	Moderately Bothered C	Very Bothered D	Extremely Bothered E
**35.** How much are you bothered by problems using your hands, arms, or legs?	☐	☐	☐	☐	☐
**36.** How much are you bothered by problems with your back?	☐	☐	☐	☐	☐
**37.** How much are you bothered by problems doing work around your home?	☐	☐	☐	☐	☐
**38.** How much are you bothered by problems with bathing, dressing, toileting, or other personal care?	☐	☐	☐	☐	☐
**39.** How much are you bothered by problems with sleep and rest?	☐	☐	☐	☐	☐
**40.** How much are you bothered by problems with leisure or recreational activities?	☐	☐	☐	☐	☐
**41.** How much are you bothered by problems with your friends, family, or other important people in your life?	☐	☐	☐	☐	☐
**42.** How much are you bothered by problems with thinking, concentrating, or remembering?	☐	☐	☐	☐	☐
**43.** How much are you bothered by problems adjusting or coping with your injury or arthritis?	☐	☐	☐	☐	☐
**44.** How much are you bothered by problems doing your usual work?	☐	☐	☐	☐	☐
**45.** How much are you bothered with feeling dependent on others?	☐	☐	☐	☐	☐
**46.** How much are you bothered with stiffness and pain?	☐	☐	☐	☐	☐
