# Supplementary material for: Pelvic ring injury in the elderly: Fragile patients with substantial mortality rates and long-term physical impairment
Source: PLoS One. 2019 May 28;14(5):e0216809. doi: 10.1371/journal.pone.0216809 (PMC6538139; doi:10.1371/journal.pone.0216809)
Supplement: S2 File — (PDF) [file pone.0216809.s002.pdf]

We zijn geïnteresseerd in hoe u deze week omgaat met de gevolgen van uw letsel(s) of aandoening(en). We willen graag weten of u hierdoor problemen ondervindt in uw dagelijkse bezigheden.

Graag elke vraag beantwoorden door het best passende antwoord aan te kruisen.

Beantwoord alstublieft alle vragen, ook de vragen die ogenschijnlijk niet van toepassing zijn op uw letsel(s) of aandoening(en).

De volgende vragen hebben betrekking op hoeveel moeite u deze week heeft met dagelijkse activiteiten als gevolg van uw letsel(s) of aandoening(en).

1 Hoeveel moeite heeft u met het in of uit een lage stoel komen?

Geen moeite	*Geringe moeite*	*Matige moeite*	*Veel moeite*	*Onmogelijk om te doen*
○	○	○	○	○

2 Hoeveel moeite heeft u met het openen van medicijnflesjes of –potjes?

Geen moeite	*Geringe moeite*	*Matige moeite*	*Veel moeite*	*Onmogelijk om te doen*
○	○	○	○	○

3 Hoeveel moeite heeft u met het doen van uw dagelijkse boodschappen of anderszins winkelen?

Geen moeite	*Geringe moeite*	*Matige moeite*	*Veel moeite*	*Onmogelijk om te doen*
○	○	○	○	○

4 Hoeveel moeite heeft u met traplopen?

Geen moeite	*Geringe moeite*	*Matige moeite*	*Veel moeite*	*Onmogelijk om te doen*
○	○	○	○	○

5 Hoeveel moeite heeft u met het maken van een stevige vuist?

Geen moeite	*Geringe moeite*	*Matige moeite*	*Veel moeite*	*Onmogelijk om te doen*
○	○	○	○	○

6 Hoeveel moeite heeft u met het in of uit de douche of bad stappen?

Geen moeite	*Geringe moeite*	*Matige moeite*	*Veel moeite*	*Onmogelijk om te doen*
○	○	○	○	○

7 Hoeveel moeite heeft u met het makkelijk in slaap vallen?

Geen moeite	*Geringe moeite*	*Matige moeite*	*Veel moeite*	*Onmogelijk om te doen*
○	○	○	○	○

8 Hoeveel moeite heeft u met bukken of knielen?

Geen moeite	*Geringe moeite*	*Matige moeite*	*Veel moeite*	*Onmogelijk om te doen*
○	○	○	○	○

9 Hoeveel moeite heeft u met het gebruik van knopen, drukknopen, haakjes of ritsen?

Geen moeite	*Geringe moeite*	*Matige moeite*	*Veel moeite*	*Onmogelijk om te doen*
○	○	○	○	○

10 Hoeveel moeite heeft u met het knippen van uw eigen vingernagels?

Geen moeite	*Geringe moeite*	*Matige moeite*	*Veel moeite*	*Onmogelijk om te doen*
○	○	○	○	○

11 Hoeveel moeite heeft u met uzelf aankleden?

Geen moeite	*Geringe moeite*	*Matige moeite*	*Veel moeite*	*Onmogelijk om te doen*
○	○	○	○	○

12 Hoeveel moeite heeft u met lopen?

Geen moeite	*Geringe moeite*	*Matige moeite*	*Veel moeite*	*Onmogelijk om te doen*
○	○	○	○	○

13 Hoeveel moeite heeft u met het in beweging komen nadat u heeft gezeten of gelegen?

Geen moeite	*Geringe moeite*	*Matige moeite*	*Veel moeite*	*Onmogelijk om te doen*
○	○	○	○	○

14 Hoeveel moeite heeft u met het zelfstandig de deur uit gaan?

Geen moeite	*Geringe moeite*	*Matige moeite*	*Veel moeite*	*Onmogelijk om te doen*
○	○	○	○	○

15 Hoeveel moeite heeft u met autorijden?

Geen moeite	*Geringe moeite*	*Matige moeite*	*Veel moeite*	*Onmogelijk om te doen*
○	○	○	○	○

16 Hoeveel moeite heeft u met het zelfstandig naar het toilet gaan?

Geen moeite	*Geringe moeite*	*Matige moeite*	*Veel moeite*	*Onmogelijk om te doen*
○	○	○	○	○

17 Hoeveel moeite heeft u met het gebruiken van knoppen of hendels (bijvoorbeeld het openen van deuren of het open draaien van autoramen)?

Geen moeite	*Geringe moeite*	*Matige moeite*	*Veel moeite*	*Onmogelijk om te doen*
○	○	○	○	○

18 Hoeveel moeite heeft u met schrijven of typen?

Geen moeite	*Geringe moeite*	*Matige moeite*	*Veel moeite*	*Onmogelijk om te doen*
○	○	○	○	○

19 Hoeveel moeite heeft u met het draaien om uw as?

Geen moeite	*Geringe moeite*	*Matige moeite*	*Veel moeite*	*Onmogelijk om te doen*
○	○	○	○	○

20 Hoeveel moeite heeft u met het uitvoeren van uw gebruikelijke lichamelijke recreatieve activiteiten, zoals fietsen, hardlopen of wandelen?

Geen moeite	*Geringe moeite*	*Matige moeite*	*Veel moeite*	*Onmogelijk om te doen*
○	○	○	○	○

21 Hoeveel moeite heeft u met het uitvoeren van uw gebruikelijke vrijetijdsbesteding, zoals hobby’s, handwerken, tuinieren, kaarten of uitgaan met vrienden?

Geen moeite	*Geringe moeite*	*Matige moeite*	*Veel moeite*	*Onmogelijk om te doen*
○	○	○	○	○

22 Hoeveel moeite heeft u met seksuele activiteiten?

Geen moeite	*Geringe moeite*	*Matige moeite*	*Veel moeite*	*Onmogelijk om te doen*
○	○	○	○	○

23 Hoeveel moeite heeft u met het verrichten van lichte huishoudelijke activiteiten of tuinwerkzaamheden, zoals afstoffen, afwassen of planten water geven?

Geen moeite	*Geringe moeite*	*Matige moeite*	*Veel moeite*	*Onmogelijk om te doen*
○	○	○	○	○

24 Hoeveel moeite heeft u met het verrichten van zware huishoudelijke activiteiten of tuinwerkzaamheden, zoals vloeren dweilen, stofzuigen of grasmaaien?

Geen moeite	*Geringe moeite*	*Matige moeite*	*Veel moeite*	*Onmogelijk om te doen*
○	○	○	○	○

25 Hoeveel moeite heeft u met het uitvoeren van uw dagelijkse werk, zoals een betaalde baan, huishouden of vrijwilligerswerk?

Geen moeite	*Geringe moeite*	*Matige moeite*	*Veel moeite*	*Onmogelijk om te doen*
○	○	○	○	○

De volgende vragen informeren naar hoe vaak u deze week problemen ervaart, die veroorzaakt worden door uw letsel(s) of aandoening(en).

26 Hoe vaak loopt u mank?

Nooit	*Zelden*	*Soms*	*Meestal*	*Altijd*
○	○	○	○	○

27 Hoe vaak vermijdt u gebruik van uw pijnlijke ledematen of rug?

Nooit	*Zelden*	*Soms*	*Meestal*	*Altijd*
○	○	○	○	○

28 Hoe vaak zit uw knie op slot of gaat u door uw knie?

Nooit	*Zelden*	*Soms*	*Meestal*	*Altijd*
○	○	○	○	○

29 Hoe vaak heeft u concentratieproblemen?

Nooit	*Zelden*	*Soms*	*Meestal*	*Altijd*
○	○	○	○	○

30 Hoe vaak beïnvloedt het te veel doen op een dag uw bezigheden van de volgende dag?

Nooit	*Zelden*	*Soms*	*Meestal*	*Altijd*
○	○	○	○	○

31 Hoe vaak gedraagt u zich geïrriteerd tegenover mensen om u heen (bijvoorbeeld mensen afsnauwen, kortaf reageren of snel bekritiseren)?

Nooit	*Zelden*	*Soms*	*Meestal*	*Altijd*
○	○	○	○	○

32 Hoe vaak bent u moe?

Nooit	*Zelden*	*Soms*	*Meestal*	*Altijd*
○	○	○	○	○

33 Hoe vaak voelt u zich lichamelijk beperkt?

Nooit	*Zelden*	*Soms*	*Meestal*	*Altijd*
○	○	○	○	○

34 Hoe vaak voelt u zich boos of gefrustreerd vanwege uw letsel(s) of aandoening(en)?

Nooit	*Zelden*	*Soms*	*Meestal*	*Altijd*
○	○	○	○	○

De volgende vragen hebben betrekking op in welke mate u <u>deze week</u> gehinderd wordt door problemen vanwege uw letsel(s) of aandoening(en).

35 In welke mate wordt u gehinderd door problemen bij het gebruik van uw armen, handen of benen?

Geen hinder	*Geringe hinder*	*Matige hinder*	*Veel hinder*	*Extreem veel hinder*
○	○	○	○	○

36 In welke mate wordt u gehinderd door rugproblemen?

Geen hinder	*Geringe hinder*	*Matige hinder*	*Veel hinder*	*Extreem veel hinder*
○	○	○	○	○

37 In welke mate wordt u gehinderd door problemen tijdens werkzaamheden rondom uw huis?

Geen hinder	*Geringe hinder*	*Matige hinder*	*Veel hinder*	*Extreem veel hinder*
○	○	○	○	○

38 In welke mate wordt u gehinderd door problemen met douchen of in bad gaan, aankleden, naar het toilet gaan of andere persoonlijke verzorging?

Geen hinder	*Geringe hinder*	*Matige hinder*	*Veel hinder*	*Extreem veel hinder*
○	○	○	○	○

39 In welke mate wordt u gehinderd door problemen met slapen en rusten?

Geen hinder	*Geringe hinder*	*Matige hinder*	*Veel hinder*	*Extreem veel hinder*
○	○	○	○	○

40 In welke mate wordt u gehinderd door problemen bij vrijetijdsbesteding en recreatieve activiteiten?

Geen hinder	*Geringe hinder*	*Matige hinder*	*Veel hinder*	*Extreem veel hinder*
○	○	○	○	○

41 In welke mate wordt u gehinderd door problemen met uw vrienden, familie of andere belangrijke mensen in uw leven?

Geen hinder	*Geringe hinder*	*Matige hinder*	*Veel hinder*	*Extreem veel hinder*
○	○	○	○	○

42 In welke mate wordt u gehinderd door problemen met nadenken, concentreren of onthouden?

Geen hinder	*Geringe hinder*	*Matige hinder*	*Veel hinder*	*Extreem veel hinder*
○	○	○	○	○

43 In welke mate wordt u gehinderd door problemen met aanpassen aan of omgaan met uw letsel(s) of aandoening(en)?

Geen hinder	*Geringe hinder*	*Matige hinder*	*Veel hinder*	*Extreem veel hinder*
○	○	○	○	○

44 In welke mate wordt u gehinderd door problemen met het doen van uw dagelijkse werk?

Geen hinder	*Geringe hinder*	*Matige hinder*	*Veel hinder*	*Extreem veel hinder*
○	○	○	○	○

45 In welke mate wordt u gehinderd door problemen met het afhankelijk voelen van anderen?

Geen hinder	*Geringe hinder*	*Matige hinder*	*Veel hinder*	*Extreem veel hinder*
○	○	○	○	○

46 In welke mate wordt u gehinderd door problemen met stijfheid en pijn?

Geen hinder	*Geringe hinder*	*Matige hinder*	*Veel hinder*	*Extreem veel hinder*
○	○	○	○	○
