# Supplementary material for: Pelvic ring injury in the elderly: Fragile patients with substantial mortality rates and long-term physical impairment
Source: PLoS One. 2019 May 28;14(5):e0216809. doi: 10.1371/journal.pone.0216809 (PMC6538139; doi:10.1371/journal.pone.0216809)
Supplement: S4 File — (PDF) [file pone.0216809.s004.pdf]

Zet bij iedere groep in de lijst hieronder een kruisje in het hokje voor de zin die het best past bij uw eigen gezondheidstoestand vandaag.

Mobiliteit
○ Ik heb geen problemen met lopen
○ Ik heb een beetje problemen met lopen
○ Ik heb matige problemen met lopen
○ Ik heb ernstige problemen met lopen
○ Ik ben niet in staat om te lopen

Zelfzorg
○ Ik heb geen problemen om mijzelf te wassen of aan te kleden
○ Ik heb een beetje problemen om mijzelf te wassen of aan te kleden
○ Ik heb matige problemen om mijzelf te wassen of aan te kleden
○ Ik heb ernstige problemen om mijzelf te wassen of aan te kleden
○ Ik ben niet in staat mijzelf te wassen of aan te kleden

Dagelijkse activiteiten (bijv. Werk, studie, huishouden, gezins- en vrijetijdsactiviteiten)
○ Ik heb geen problemen met mijn dagelijkse activiteiten
○ Ik heb een beetje problemen met mijn dagelijkse activiteiten
○ Ik heb matige problemen met mijn dagelijkse activiteiten
○ Ik heb ernstige problemen met mijn dagelijkse activiteiten
○ Ik ben niet in staat mijn dagelijkse activiteiten uit te voeren

Pijn/ongemak
○ Ik heb geen pijn of ongemak
○ Ik heb een beetje pijn of ongemak
○ Ik heb matige pijn of ongemak
○ Ik heb ernstige pijn of ongemak
○ Ik heb extreme pijn of ongemak

Stemming
○ Ik ben niet angstig of somber
○ Ik ben een beetje angstig of somber
○ Ik ben matig angstig of somber
○ Ik ben erg angstig of somber
○ Ik ben extreem angstig of somber
